# Urinary Epithelial Cell Adhesion Molecule (EpCAM) as a Noninvasive Biomarker for Detecting Clinically Significant Prostate Cancer in Men With Equivocal PSA Levels

**DOI:** 10.1155/bmri/3839816

**Published:** 2026-03-16

**Authors:** Bismark Opoku Mensah, Linda Ahenkorah Fondjo, Paul Nsiah, Emma Edwards

**Affiliations:** ^1^ Department of Biological Sciences, University of Worcester, Worcester, UK, worcester.ac.uk; ^2^ Department of Molecular Medicine, KNUST School of Medicine and Dentistry, Kumasi, Ghana; ^3^ Department of Chemical Pathology, University of Cape Coast Medical school, Cape Coast, Ghana

**Keywords:** ISUP grade group, prostate cancer, PSA, PSA density, urinary biomarker, urinary creatinine, urinary EpCAM

## Abstract

**Background:**

Prostate‐specific antigen (PSA) is widely used for prostate cancer screening, but its limited specificity in the diagnostic “grey zone” (4–10 ng/mL) results in unnecessary biopsies. This study evaluated urinary epithelial cell adhesion molecule (EpCAM), normalised to urinary creatinine, as a noninvasive biomarker for identifying clinically significant prostate cancer (csPCa) in men with equivocal PSA levels.

**Methods:**

This cross‐sectional study included 286 men with PSA levels of 4–10 ng/mL scheduled for prostate biopsy. Histopathological outcomes were classified using ISUP Grade Groups. Urinary EpCAM was quantified by ELISA, and diagnostic performance for csPCa was assessed using nonparametric analyses, ROC curves and logistic regression.

**Results:**

Urinary EpCAM/Cr levels increased significantly across diagnostic categories and were highest in men with csPCa (*p* < 0.001). EpCAM/Cr showed strong discrimination for csPCa (AUC = 0.816), outperforming serum PSA and PSA density. In multivariable analysis, EpCAM/Cr (aOR = 1.07; 95% CI: 1.02–1.14; *p* = 0.009) and PSA density (aOR = 1.81; 95% CI: 1.20–3.18; *p* = 0.013) were independent predictors of csPCa, and EpCAM/Cr correlated positively with tumour aggressiveness (*ρ* = 0.609; *p* < 0.001).

**Conclusion:**

Urinary EpCAM/Cr shows promise as a noninvasive biomarker for detecting csPCa in men with PSA levels in the diagnostic grey zone.

## 1. Introduction

Prostate cancer is one of the most prevalent cancers among men and a major contributor to global cancer‐related mortality [1]. Early detection is critical for improving prognosis and guiding treatment decisions. However, the current dependence on serum prostate‐specific antigen (PSA) testing is limited by suboptimal specificity [2, 3].

PSA is prostate‐specific rather than cancer‐specific, and may be elevated in several benign prostatic conditions [4]. This limitation is particularly evident within the diagnostic “grey zone” (4–10 ng/mL), where PSA fails to reliably distinguish malignant from benign prostate conditions [5], leading to unnecessary biopsies and missed diagnoses of clinically significant prostate cancer (csPCa) [6].

The epithelial cell adhesion molecule (EpCAM) is a membrane‐bound glycoprotein that mediates interactions between epithelial cells and regulates their proliferation and differentiation [7]. Overexpression of EpCAM has been reported in several epithelial malignancies, including prostate cancer [8, 9]. Proteolytic cleavage of EpCAM releases soluble extracellular fragments into biological fluids such as blood and urine [10]. These soluble fragments have been proposed as a potential biomarker for tumor detection and disease activity [11, 12].

Despite growing global interest in EpCAM‐based assays, the clinical utility of urinary EpCAM for identifying csPCa remains inadequately defined in African populations. Most biomarker discovery and validation studies have been conducted in European, North American or Asian cohorts, with limited representation of African men [13, 14].

This study therefore evaluated the diagnostic performance of urinary EpCAM, normalised to urinary creatinine in differentiating csPCa from benign and indolent disease among men with PSA concentrations within the diagnostic “grey zone”.

## 2. Materials and Methods

### 2.1. Study Design and Participants

This cross‐sectional study was conducted at the 37 Military Hospital in Ghana between February 2019 and August 2022. Male participants aged ≥ 45 years with PSA levels within the 4–10 ng/mL interval who were referred for prostate biopsy were consecutively recruited. Each participant underwent multiparametric magnetic resonance imaging (mpMRI). This was followed by both systematic and targeted prostate biopsies performed in accordance with standard clinical practice.

### 2.2. Eligibility Criteria

Men were eligible for inclusion if they were aged 45 years or older and had a serum PSA concentration between 4 and 10 ng/mL. Serum PSA concentrations were obtained from routine clinical laboratory results as part of standard diagnostic evaluation. Eligibility required a total PSA value between 4 and 10 ng/mL measured within 2 weeks prior to the scheduled prostate biopsy. Participants were excluded if they had a prior diagnosis of prostate cancer, a history of prostate surgery or therapy or evidence of active or recent urinary tract infection. Additional exclusions included urological catheterisation or instrumentation within the preceding 2 weeks or at enrolment, known or suspected malignancies of the bladder, kidney or ureter, and any other epithelial malignancy diagnosed within the preceding 2 years. Men presenting with either macroscopic or microscopic haematuria, or with chronic inflammatory urological conditions, were also excluded from the study.

### 2.3. Sample Size Determination

The required sample size was calculated using the precision‐based approach [15, 16], assuming an expected sensitivity of 85% and specificity of 80% for urinary EpCAM in detecting prostate cancer. A prostate cancer prevalence of 24% was adopted based on previous studies conducted in this population [17–19]. With a 95% confidence level and a desired precision of ± 10%, the minimum estimated sample size was 286 participants. A total of 328 men were screened during the study period, of whom 286 met the eligibility criteria and provided informed consent, resulting in a participation rate of 87.2%.

### 2.4. Ethical Consideration

Approval for this study was obtained from the 37 Military Hospital Ethics Committee (37MH‐IRB IP/306/2019). Prior to enrolment, written informed consent was obtained from all participants. All procedures were carried out in line with the ethical guidelines of the Declaration of Helsinki.

### 2.5. Collection of Urine Samples

Urine samples were collected immediately after a standardised digital rectal examination (DRE) performed by certified urologists in accordance with an institutional clinical protocol. Although DREs were conducted by more than one clinician, all followed the same standardised procedure to minimise interoperator variability.

First‐void urine (10–20 mL) was obtained into sterile containers preloaded with protease inhibitor (Roche Diagnostics, Germany) to preserve protein integrity. Samples were transported on ice to the laboratory within 1 h of collection.

Each specimen was centrifuged at 1500 × g for 10 min at 4°C to remove cell debris. The supernatant was aliquoted into cryovials and stored at −80°C until biochemical analysis. All samples were subjected to a single freeze–thaw cycle prior to assay, as repeated freeze–thaw events are known to adversely affect protein stability and quantitative reliability in biomarker assays [20].

### 2.6. Laboratory Analyses

#### 2.6.1. Measurement of Urinary Creatinine

Urine creatinine concentrations were measured in duplicate using the Jaffe colorimetric method on an automated chemistry analyser (Beckman Coulter AU480, United States). The assay had a detection range of 0.0078–4.2 mg/mL, with an intra‐assay coefficient of variation of less than 5%. Measured creatinine concentrations were used to normalize urinary EpCAM levels to correct for variations in urine concentration among samples.

#### 2.6.2. Measurement of Urinary EpCAM

Urinary EpCAM concentrations were measured using a commercial sandwich ELISA kit (Abcam, United Kingdom) in accordance with the manufacturer′s instructions. Recombinant human EpCAM standards provided with the kit were reconstituted and serially diluted in standard diluent to construct a calibration curve. The assay had a lower limit of detection of 0.03 ng/mL and a recovery rate of 92.8%. The intra‐assay and interassay coefficients of variation were 8.99% and 12.28%, respectively. Each urine sample was assayed in duplicate, and absorbance was measured at 450 nm using a microplate reader (BioTek Synergy HT, United States). EpCAM concentrations were calculated by interpolation from the standard curve generated using a four‐parameter logistic regression model.

### 2.7. Normalisation of EpCAM to Urinary Creatinine

Urine EpCAM concentrations (nanograms per millilitre) were normalised to urinary creatinine concentrations (milligrams per millilitre) measured from the same sample. This was done to account for variations in urine volumes and urine flow rates. The normalised EpCAM concentration was expressed as nanograms of EpCAM per milligram of creatinine (ng/mg Cr) and calculated using the formula: EpCAM_norm_ = EpCAM concentration (nanograms/millilitre)/urinary creatinine (milligrams per millitre).

### 2.8. Clinical Reference Standard

Histopathology evaluation of systematic and targeted prostate biopsy cores served as the diagnostic reference standard. All biopsy specimens were examined and graded by a certified pathologist in accordance with the International Society of Urological Pathology (ISUP) grading system. csPCa was defined as ISUP Grade Group ≥ 2.

### 2.9. Statistical Analysis

Data were analysed using IBM SPSS Statistics Version 27.0 (IBM Corp., Armonk, New York, United States). Normally distributed variables were summarised as means ± standard deviation and compared using one‐way ANOVA. The Kruskal–Wallis test was used to analyse non‐normally distributed variables. Post hoc pairwise comparisons were done with the Mann–Whitney *U* test.

Associations between urinary EpCAM/Cr and ISUP grade were examined using Spearman′s rank correlation. Diagnostic accuracy was evaluated using ROC curves, and optimal thresholds were identified through the Youden index. Positive and negative predictive values (PPV and NPV) were computed using the observed prevalence of csPCa in the study cohort. For multivariable and dual‐marker models, ROC analyses were performed using the predicted probabilities generated from logistic regression. Optimal cutoff thresholds were determined from the ROC coordinates using maximisation of the Youden index.

To determine which variables could predict csPCa, logistic regression models were employed. Variables that showed a univariable association at *p* < 0.10 were subsequently considered in multivariable analyses. Multicollinearity was assessed using variance inflation factors (VIF). Given the mathematical dependence of prostate‐specific antigen density (PSAD) on PSA and PSAD were not included together in the same multivariable model. The accuracy of the final regression model was evaluated using the Hosmer–Lemeshow goodness of fit test. A two‐sided *p* < 0.05 was considered statistically significant.

## 3. Results

### 3.1. Baseline Demographic and Clinical Characteristics

A total of 286 men with serum PSA levels between 4 and 10 ng/mL who were scheduled for prostate biopsy were enrolled in the study. Of these, 28 (9.8%) were histologically classified as normal, 132 (46.2%) as benign prostatic conditions, 52 (18.2%) as indolent prostate cancer (ISUP Grade Group 1) and 74 (25.9%) as csPCa (ISUP Grade Group ≥ 2).

There were no statistically significant differences among the groups for age, body mass index (BMI), or diastolic blood pressure (DBP) (*p* = 0.916, 0.573 and 0.255, respectively). Systolic blood pressure (SBP) differed significantly across groups (*p* = 0.029), with the highest mean values recorded among participants with normal prostate histology.

Serum PSA concentrations increased progressively across the groups, from a median of 5.9 ng/mL (IQR: 4.6–7.2) in men with nonmalignant prostate conditions to 9.3 ng/mL (IQR: 4.8–9.7) in those with csPCa (*p* = 0.031). Prostate volume showed an inverse trend, decreasing from 66.3 cm^3^ (IQR: 57.9–74.7) in men with nonmalignant prostate histology to 37.3 cm^3^ (IQR: 30.8–46.9) in those with csPCa (*p* < 0.001). PSAD also differed significantly among the groups (*p* < 0.001), with the highest median values (0.26, IQR: 0.20–0.33) in men with csPCa.

Urinary creatinine concentrations were comparable across groups (*p* = 0.440). In contrast, both urinary EpCAM concentrations and EpCAM/Cr differed significantly (*p* < 0.001 for both), showing a stepwise increase from normal to malignant histology. Median urinary EpCAM increased from 0.7 ng/mL (IQR: 0.4–1.1) in men with normal prostates to 3.0 ng/mL (IQR: 0.9–6.2) in those with csPCa. The corresponding EpCAM/Cr rose from 0.8 ng/mg Cr (IQR: 0.3–1.3) in the normal group to 3.0 ng/mg Cr (IQR: 0.8–5.5) in the csPCa group (Table 1).

**Table 1:** Baseline demographic and clinical characteristics of study participants.

Variable	Normal prostate (*n* = 28)	Benign conditions (*n* = 132)	ISUP Grade Group 1 (*n* = 52)	*I* *S* *U* *P* *G* *r* *a* *d* *e* *G* *r* *o* *u* *p* ≥ 2(*n* = 74)	*p* value
Age (years)	60.5 ± 9.0	61.1 ± 8.9	61.8 ± 11.0	61.8 ± 10.4	0.916
Systolic BP (mmHg)	137.5 ± 21.5	132.1 ± 18.7	131.5 ± 19.3	126.2 ± 15.5	**0.029**
Diastolic BP (mmHg)	87.7 ± 13.8	83.73 ± 12.6	82.9 ± 13.4	82.1 ± 11.5	0.255
BMI (kg/m^2^)	24.9 (23.0–30.3)	26.9 (23.6–31.6)	25.8 (22.3–30.6)	27.0 (22.8–30.1)	0.573
PSA (ng/mL)	5.9 (4.6–7.2)	8.2 (5.5–8.4)	7.8 (4.9–8.7)	9.3 (4.8–9.7)	**0.031**
Prostate volume (cm^3^)	66.3 (57.9–74.7)	76.4 (58.8–84.0)	50.6 (45.3–63.5)	37.3 (30.8–46.9)	**< 0.001**
PSA density (ng/mL/cm^3^)	0.09 (0.06–0.12)	0.12 (0.08–0.16)	0.20 (0.15–0.24)	0.26 (0.20–0.33)	**< 0.001**
Urinary creatinine (mg/mL)	1.3 (0.8–1.7)	1.2 (0.8–1.7)	1.1 (0.6–1.5)	1.0 (0.8–2.2)	0.440
Urinary EpCAM (ng/mL)	0.7 (0.4–1.1)	1.0 (0.5–1.4)	1.9 (0.8–4.6)	3.0 (0.9–6.2)	**< 0.001**
Urinary EpCAM/Cr (ng/mg Cr)	0.8 (0.3–1.3)	0.8 (0.3–1.6)	1.7 (0.7–5.2)	3.0 (0.8–5.5)	**< 0.001**

*Note:* Data are presented as mean ± standard deviation(SD) and median (interquartile range). Bolded *p* values are significant.

Abbreviations: BP, blood pressure; BMI, body mass index; EpCAM, epithelial cell adhesion molecule; ISUP, International Society of Urological Pathology; PSA, prostate‐specific antigen.

Pairwise comparisons showed that both urinary EpCAM and EpCAM/Cr were significantly higher in csPCa (ISUP Grade Group ≥ 2) compared with indolent prostate cancer (ISUP Grade Group 1) (*p* = 0.012 and *p* = 0.003, respectively). No significant differences were observed between the normal and benign groups (*p* = 0.189 and *p* = 0.730, respectively) (Figure 1).

Figure 1Box‐and‐whisker plots showing urinary EpCAM and EpCAM/Cr levels across diagnostic categories. Boxes represent the interquartile range, horizontal lines within boxes indicate median values. Circles (○) represent outliers, and asterisks ( ^∗^) denote extreme outliers.

**Figure:**
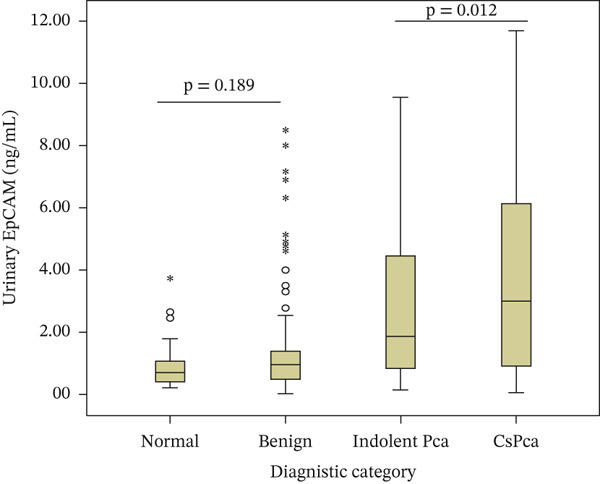
(a) Urinary EpCAM levels across diagnostic categories

**Figure:**
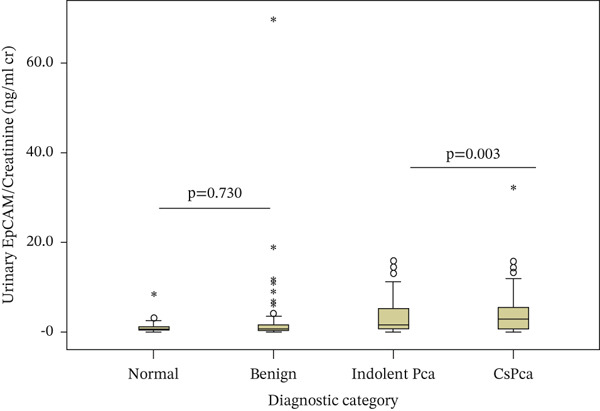
(b) Urinary EpCAM/Cr levels across diagnostic categories

### 3.2. Diagnostic Performance of Urinary EpCAM/Cr, PSA, PSAD and Combined Models

Among the individual markers, urinary EpCAM/Cr demonstrated the best performance, with an area under the curve (AUC) of 0.816 (95% CI: 0.751–0.838; *p* < 0.001), achieving a sensitivity of 88.4% and specificity of 90.9% at an optimal cutoff value of 1.7 ng/mg Cr. In comparison, serum PSA and PSAD exhibited limited diagnostic accuracy, with AUC values of 0.472 (95% CI: 0.392–0.553; *p* = 0.337) and 0.462 (95% CI: 0.395–0.540; *p* = 0.480), respectively. Although both parameters demonstrated good sensitivities, their specificities were low.

The combined model incorporating urinary EpCAM/Cr, PSA and PSAD achieved a comparable performance to the urinary EpCAM/Cr alone (*p* = 0.091), with an AUC of 0.825 (95% CI: 0.768–0.841, *p* < 0.001), and a sensitivity and specificity of 86.9% and 91.7%, respectively, at a cutoff of 0.24.

**Figure 2:**
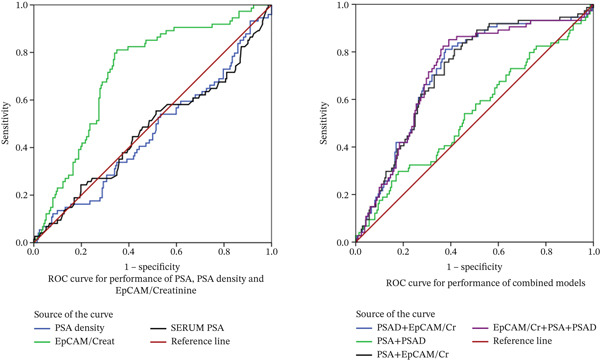
ROC curves showing the diagnostic performance of urinary EpCAM‐to‐creatinine ratio (EpCAM/Cr), PSA, PSA density and the combined models.

When dual‐marker combinations were evaluated, the PSAD + EpCAM/Cr model achieved the best overall diagnostic performance, with an AUC of 0.715 (95% CI: 0.648–0.731; *p* < 0.001), sensitivity of 83.8% and specificity of 60.8%. The PSA + EpCAM/Cr combination produced a similar AUC of 0.712 (95% CI: 0.686–0.738; *p* < 0.001), with sensitivity and specificity of 93.2% and 68.0%, respectively. Conversely, the PSA + PSAD model demonstrated poor discriminative capacity (AUC = 0.538; 95% CI: 0.459–0.586; *p* = 0.333), despite its high sensitivity of 95.9% (Table 2 and Figure 2).

**Table 2:** Diagnostic performance of urinary EpCAM/Cr, PSA, PSA density and the combined models in distinguishing clinically significant prostate cancer.

Parameter	Cutoff	Sensitivity (%)	Specificity (%)	PPV (%)	NPV (%)	AUC (95% CI)	*p* value
Urinary EpCAM/Cr (ng/mg Cr)	1.7	88.4	90.9	77.4	95.7	0.816 (0.751 –0.838)	**< 0.001**
PSA (ng/mL)	6.7	89.7	35.1	32.5	90.6	0.472 (0.392 –0.553)	0.337
PSA density (ng/mL/cm^3^)	0.17	81.1	51.4	36.8	88.7	0.462 (0.395 –0.540)	0.480
EpCAM/Cr + PSA + PSAD	0.24	86.9	91.7	78.7	95.2	0.825 (0.768–0.841)	**< 0.001**
PSAD + EpCAM/Cr	0.19	83.8	60.8	42.8	91.5	0.715 (0.648–0.731)	**< 0.001**
PSA + PSAD	0.21	95.9	20.3	29.6	93.4	0.538 (0.459–0.586)	0.333
PSA + EpCAM/Cr	0.18	93.2	68.0	50.5	96.5	0.712 (0.686–0.738)	**< 0.001**

*Note:* Bolded *p* values are significant.

Abbreviations: AUC, area under the curve; NPV, negative predictive value; PPV, positive predictive value;PSA, prostate‐specific antigen; PSAD, prostate‐specific antigen density.

### 3.3. Predictors of csPCa

Univariable logistic regression analysis identified urinary EpCAM/Cr and PSAD as significant predictors of clinically csPCa. Urinary EpCAM/Cr was associated with increased odds of csPCa (OR = 1.08; 95% CI: 1.02–1.14; *p* = 0.008), whereas PSAD also demonstrated a significant association (OR = 2.20; 95% CI: 1.12–4.30; *p* = 0.030). In contrast, age, serum PSA and prostate volume showed no significant associations with csPCa (*p* = 0.662, 0.570 and 0.600, respectively). All variables demonstrated acceptable collinearity, with VIF values ranging from 1.003 to 2.893.

In multivariable Model 1, which included age, PSA, prostate volume and urinary EpCAM/Cr, the urinary EpCAM/Cr remained the only independent predictor of csPCa (aOR = 1.07; 95% CI: 1.02–1.14; *p* = 0.009). Age (*p* = 0.677), PSA (*p* = 0.675) and prostate volume (*p* = 0.983) were not independently associated with csPCa. In multivariable Model 2, which incorporated age, PSAD and urinary EpCAM/Cr, both urinary EpCAM/Cr (aOR = 1.07; 95% CI: 1.02–1.14; *p* = 0.008) and PSAD (aOR = 1.81; 95% CI: 1.20–3.18; *p* = 0.013) remained independent predictors of csPCa (Table 3).

**Table 3:** Logistic regression analyses for predictors of clinically significant prostate cancer.

Variable	Univariable OR (95% CI)	*p* value	Multivariable model 1^a^ (*P* *S* *A* + *p* *r* *o* *s* *t* *a* *t* *e* *v* *o* *l* *u* *m* *e* + *E* *p* *C* *A* *M*/*C* *r*) aOR (95% CI)	*p* value	Multivariable model 2^b^ (*P* *S* *A* *D* + *E* *p* *C* *A* *M*/*C* *r*) aOR (95% CI)	*p* value
Age (years)	1.01 (0.98–1.03)	0.662	1.01 (0.978–1.03)	0.677	1.01(0.98–1.03)	0.677
PSA (ng/mL)	0.96 (0.83–1.11)	0.570	0.97 (0.83–1.13)	0.675	—	—
Prostate Volume (cm^3^)	1.00 (0.99–1.01)	0.600	1.00 (0.98–1.01)	0.983	—	—
PSA density (ng/mL/cm^3^)	2.20 (1.12–4.30)	**0.030**	—	—	1.81 (1.20–3.18)	**0.013**
Urinary EpCAM/Cr (ng/mg Cr)	1.08(1.02–1.14)	**0.008**	1.07 (1.02–1.14)	**0.009**	1.07 (1.02–1.14)	**0.008**

*Note:* Bolded *p* values are significant.

^a^Model 1: (Age + serum PSA + prostate volume + urinary EpCAM/Cr); Hosmer–Lemeshow goodness of fit *p* = 0.64. AUC = 0.864 (95% CI: 0.786–0.928).

^b^Model 2: (Age + PSA density + urinary EpCAM/Cr); Hosmer–Lemeshow goodness of fit *p* = 0.48. AUC = 0.811 (95% CI: 0.793–0.910).

### 3.4. Distribution and Correlation of Urinary EpCAM/Cr Across ISUP Grade Groups

**Figure 3:**
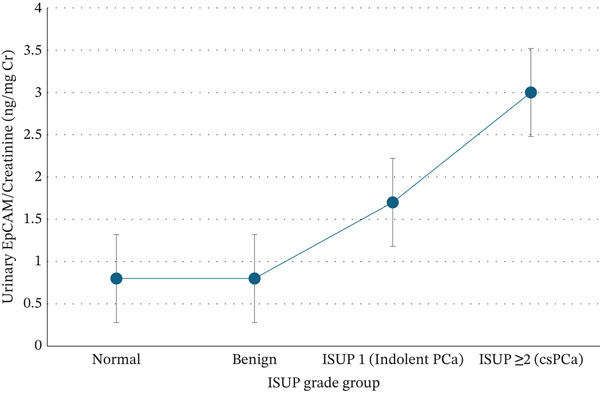
Median urinary EpCAM/Cr (ng/mg Cr) across ISUP grade groups.

The Spearman′s rank correlation analysis was used to evaluate the relationship between urinary EpCAM/Cr levels and tumour aggressiveness. A significant positive correlation was observed between urinary EpCAM/Cr levels and ISUP grade (*ρ* = 0.609; *p* < 0.001). Urinary EpCAM/Cr levels demonstrated an increase across the diagnostic categories, from normal and benign prostates to indolent (ISUP Grade Group 1) and csPCa (ISUP Grade Group ≥ 2) (Figure 3).

*Values represent medians and interquartile ranges.*

## 4. Discussion

This study evaluated the diagnostic utility of urinary epithelial cell adhesion molecule normalised to creatinine (EpCAM/Cr) in distinguishing csPCa from benign and indolent disease among men with serum PSA levels within the diagnostic “grey zone” (4–10 ng/mL).

Serum PSA levels increased progressively from men with normal prostate histology to those with csPCa, corroborating earlier reports of a modest positive association between PSA and prostate pathology [21–23]. Prostate volume showed an inverse trend, decreasing as cancer grade increased (Table 1). This likely reflects loss of glandular tissue and peripheral zone volume during cancer development [24, 25]. As a result, PSAD increased across disease categories, consistent with reports that higher PSAD may indicate greater tumour burden and aggressiveness [26].

Urinary EpCAM/Cr levels increased markedly with tumour grade, showing nearly a fourfold elevation in men with csPCa compared with those with benign and normal histology. This suggests a degree of specificity of EpCAM expression in malignant transformation in the prostate gland rather than in inflammatory or hyperplastic processes [27]. Pairwise comparisons further showed significantly higher urinary EpCAM/Cr levels in csPCa relative to indolent tumours. These findings are consistent with earlier studies by Benko et al. and Dai et al. who reported increased EpCAM expression in prostate cancer cells and its potential involvement in tumour progression [28, 29]. These results suggest a biological link between EpCAM expression and prostate cancer severity [30], supporting the biomarker′s potential to distinguish clinically significant disease from indolent or benign pathology. EpCAM also undergoes regulated proteolytic cleavage, releasing extracellular fragments that can be detected in biological fluids [31]. Increased epithelial turnover, tumour cell shedding and disruption of glandular integrity in prostate cancer may therefore facilitate the release of EpCAM or EpCAM‐derived fragments into urine, particularly following prostatic manipulation. These biological features provide a plausible mechanistic basis for the observed association between elevated urinary EpCAM levels and csPCa in this study.

ROC curve analysis also demonstrated that urinary EpCAM/Cr was the most reliable indicator of csPCa compared with serum PSA and PSAD. This finding reinforces the hypothesis that urine molecular markers derived from tumour epithelium offer greater diagnostic specificity than serum‐based surrogates such as PSA or PSAD [32, 33]. Recent evidence further supports the diagnostic potential of urinary EpCAM in prostate cancer detection as reported by Yuan et al. [34]. The performance of the combined model incorporating EpCAM/Cr, PSA and PSAD was comparable with the EpCAM/Cr alone (AUC of 0.825 vs. 0.816), suggesting the latter′s robustness as a stand‐alone biomarker. The AUC for PSA and PSAD‐based models (Table 2) was associated with wider confidence intervals, indicating limited precision. This likely reflects the intrinsic diagnostic limitations of PSA and PSAD.

There was no significant association between age and csPCa in this study, a finding that departs from classical risk models of prostate cancer [35–37]. This finding is likely attributable to the relatively narrow or homogeneous age distribution among participants in this study, which may have constrained between‐group variability and reduced statistical power to detect age‐related effects [38].

Furthermore, the absence of significant associations for serum PSA and prostate volume aligns with contemporary evidence suggesting that these conventional clinical markers demonstrate limited discriminative ability within the PSA ‘grey zone’ [39].

In this study, urinary EpCAM/Cr emerged as a consistent and independent predictor of csPCa. Its persistence as a significant variable in both univariable and multivariable logistic regression models highlights its potential as a biologically robust biomarker that may reflect tumour‐specific epithelial activity [9, 40], independent of traditional clinical parameters such as age, serum PSA concentration and prostate volume.

The positive correlation observed between urinary EpCAM/Cr and ISUP grade further highlights a potential link between urinary EpCAM excretion and tumour aggressiveness. EpCAM/Cr rises from benign and indolent cases to higher‐grade prostate cancers, suggesting that EpCAM shedding into urine may reflect progressive disruption of epithelial integrity and increased tumour activity [7, 41]. This finding is consistent with histological evidence of upregulated EpCAM expression in poorly differentiated prostate adenocarcinomas [42]. In prostate tumours, EpCAM overexpression has been associated with enhanced proliferative signalling and loss of normal epithelial architecture that contributes to tumour aggressiveness and metastatic potential [43, 44]. These observations position EpCAM/Cr as a potential diagnostic biomarker and a tool for risk stratification.

From a clinical perspective, urinary EpCAM/Cr could be incorporated into existing diagnostic workflows as a noninvasive adjunct to current risk stratification strategies. In men with PSA levels within the diagnostic grey zone, EpCAM/Cr measurement could be used prior to biopsy to refine risk assessment alongside established clinical parameters. In settings where multiparametric MRI is available, EpCAM/Cr may also serve as a complementary biomarker to support biopsy decision‐making, particularly in cases with equivocal MRI findings.

These findings highlight the promising diagnostic value of urinary EpCAM/Cr for identifying csPCa in men within the PSA grey zone. However, some limitations should be considered when interpreting the results. The cross‐sectional design limits assessment of temporal causality. In addition, although multivariable models were applied, subclinical inflammatory states were not specifically assessed and may influence epithelial shedding or urinary biomarker concentrations.

This study did not include direct comparisons with other established urinary biomarkers, such as PCA3 or SelectMDx, as the primary objective was to evaluate the stand‐alone diagnostic performance of urinary EpCAM within an equivocal PSA range. Furthermore, potential preanalytical influences on urinary EpCAM levels, such as diurnal variation, dietary factors and medication use, were not systematically evaluated, although standardised collection and processing protocols were applied across all participants.

Despite these limitations, the strength and consistency of the observed associations across analytical models support the robustness of urinary EpCAM/Cr as a candidate noninvasive biomarker for csPCa. Future longitudinal and multicentre studies are needed to confirm external validity and explore the integration of urinary EpCAM/Cr into existing risk‐stratification models to support biopsy decision‐making.

## 5. Conclusion

This study demonstrates that urinary EpCAM, normalised to creatinine, shows strong potential as a noninvasive biomarker for identifying csPCa in men with serum PSA levels within the diagnostic ‘grey zone’. Urinary EpCAM/Cr effectively discriminated clinically significant malignancy from benign and indolent disease, with high sensitivity and specificity, and remained an independent predictor of csPCa in multivariable analysis.

Given its association with tumour aggressiveness and its superior discriminative performance relative to PSA‐based markers, urinary EpCAM/Cr could serve as a clinically meaningful adjunct for biopsy triage. Incorporating this marker into prebiopsy assessment pathways could reduce unnecessary biopsies among men with a low likelihood of clinically significant disease while facilitating earlier identification of individuals who stand to benefit from timely clinical intervention.

## Funding

No funding was received for this manuscript.

## Conflicts of Interest

The authors declare no conflict of interest.

## Data Availability

The data that support the findings of this study are available from the corresponding author upon reasonable request.
